# Per- and Polyfluoroalkyl Substances (PFAS) in Food Products by Liquid Chromatography Tandem Mass Spectrometry (LC-MS/MS), AOAC Official Method 2025.07, First Action

**DOI:** 10.1093/jaoacint/qsag014

**Published:** 2026-02-27

**Authors:** Toshiya Matsubara, Yusuke Mizuno, Eishi Imoto, Nozomi Maeshima, Manami Kobayashi, Okiyuki Kunihiro, William Lipps

**Affiliations:** Shimadzu Scientific Instruments, Inc, 7102 Riverwood Dr, Columbia, MD 21046, USA; Shimadzu Scientific Instruments, Inc, 7102 Riverwood Dr, Columbia, MD 21046, USA; Shimadzu Scientific Instruments, Inc, 7102 Riverwood Dr, Columbia, MD 21046, USA; Shimadzu Corporation, Shimadzu Tokyo Innovation Plaza, 3 Chome-25-40 Tonomachi, Kawasaki Ward, Kawasaki, Kanagawa 210-0821, Japan; Shimadzu Corporation, Shimadzu Tokyo Innovation Plaza, 3 Chome-25-40 Tonomachi, Kawasaki Ward, Kawasaki, Kanagawa 210-0821, Japan; Shimadzu Corporation, Shimadzu Tokyo Innovation Plaza, 3 Chome-25-40 Tonomachi, Kawasaki Ward, Kawasaki, Kanagawa 210-0821, Japan; Shimadzu Scientific Instruments, Inc, 7102 Riverwood Dr, Columbia, MD 21046, USA

## Abstract

**Background:**

Poly- and perfluoroalkyl substances (PFAS) are fluorinated organic chemicals, which include perfluorooctanoic acid (PFOA) and perfluorooctanesulfonic acid (PFOS). PFAS are persistent and bioaccumulate. PFAS can enter the food supply through plants and animals grown, raised, or processed in contaminated areas.

**Objective:**

This single-laboratory validation (SLV) study was conducted for a liquid chromatography tandem mass spectrometry (LC-MS/MS) method to determine 30 PFAS in a variety of foods, including produce, coffee, milk, protein powders, eggs, seafood, fish meat, edible offal, fish oil, baby food, and pet food, and to compare with AOAC INTERNATIONAL *Standard Method Performance Requirement* (SMPR^®^) 2023.003.

**Method:**

Matrixes were extracted using acetonitrile and QuEChERS reagents and then purified by solid-phase extraction (SPE) and concentrated under nitrogen. Fish meat did not require concentration under nitrogen, and fish oil and edible offal did not require QuEChERS reagents and sample concentration to reach the required limit of quantitation (LOQ).

**Results:**

The LC-MS/MS system provides linear responses in the range of 0.01–25 μg/kg for most target analytes, depending on the matrix. Triplicate determinations over at least three spiking concentrations were made in each matrix with quantitation by isotopic dilution. We evaluated recovery, precision, retention time, blanks, ion ratios, and signal-to-noise ratio of the qualifier ions in all matrixes to determine the LOQ. Potential interference from cholic acids were separated chromatographically.

**Conclusions:**

The method developed and validated for the analysis of PFAS in food met the LOQ and recovery and precision requirements of the SMPR.

**Highlights:**

The LC-MS/MS method developed uses a single set of operating conditions, enabling easy and rapid transfer of the method. The extraction is simplified, with only small variations necessary due to the sample matrix. The implementation of this method is cost-effective to laboratories due to minimal solvent usage and labor required to extract samples.

Poly- and perfluoroalkyl substances (PFAS) are fluorinated organic chemicals, which include perfluorooctanoic acid (PFOA) and perfluorooctanesulfonic acid (PFOS). PFAS are persistent (they do not easily break down in the environment) and bioaccumulate (they build up over time in the blood and organs). Of all the PFAS, PFOA and PFOS have been the most studied. PFOA and PFOS have been used in the manufacturing of carpet, clothing, shoes, cookware, packaging, oil and water repellents, furniture, takeout food containers, electroplating, and countless other applications. PFAS have also been used by the U.S. Department of Defense (DoD) for fire extinguishing at military base airfields at locations throughout the United States.

LC-MS/MS methods, EPA Method 537, 537.1, 533, 1633, ASTM D7979, D8421, D7968, and D8535 (1–7) have been developed for the analysis of PFAS in environmental samples. U.S. Food and Drug Administration (FDA) C-01.02 and United States Department of Agriculture (USDA) CLG—PFAS 2.03 (8–9) methods have been single-laboratory validated (SLV) for PFAS in various food products, and recently FDA has published C-010.03 (10), a SLV method for 30 PFAS in 9 matrixes. However, none of these methods have validated all 30 PFAS compounds in 11 matrixes.

The study describes a SLV of a new method to meet the guidelines provided in AOAC *Standard Method Performance Requirements* (SMPR^®^) 2023.003 (11) on the 30 PFAS shown in Table 1 in produce, coffee, milk (liquid), dairy powders and plant-based protein powders, eggs, seafood (crustaceans and mollusks), fish meat and meat of other terrestrial animals (raw, cooked, processed), edible offal of terrestrial animals, fish oil, foods for infants and young children (baby food), and pet food and animal feed and is intended for inclusion as a First Action Official Method for Analysis (OMA) and for Final Action after a collaborative study.

**Table 1.** Target analytes

Name	Abbreviation	CAS No.
Perfluorobutanoic acid	PFBA	375–22-4
Perfluoropentanoic acid	PFPeA	2706–90-3
Perfluorohexanoic acid	PFHxA	307–24-4
Perfluoroheptanoic acid	PFHpA	375–85-9
Perfluorooctanoic acid	PFOA	335–67-1
Perfluorononanoic acid	PFNA	375–95-1
Perfluorodecanoic acid	PFDA	335–76-2
Perfluoroundecanoic acid	PFUnA	2058–94-8
Perfluorododecanoic acid	PFDoA	307–55-1
Perfluorotridecanoic acid	PFTrDA	72629–94-8
Perfluorotetradecanoic acid	PFTeDA	376–06-7
Perfluorobutanesulfonic acid	PFBS	375–73-5
Perfluoropentansulfonic acid	PFPeS	2706–91-4
Perfluorohexanesulfonic acid	PFHxS	355–46-4
Perfluoroheptanesulfonic acid	PFHpS	375–92-8
Perfluorooctanesulfonic acid	PFOS	1763–23-1
Perfluorononanesulfonic acid	PFNS	68259–12-1
Perfluorodecanesulfonic acid	PFDS	335–77-3
Perfluoroundecanesulfonic acid	PFUnDS	749786–16-1
Perfluorododecanesulfonic acid	PFDoS	79780–39-5
Perfluorotridecanesulfonic acid	PFTrDS	791563–89-8
Perfluorooctanesulfonamide	PFOSA	754–91-6
9-Chlorohexadecafluoro-3-oxanonane-1-sulfonic acid	9Cl-PF3ONS	756426–58-1
11-Chloroeicosafluoro-3-oxaundecane-1-sulfonic acid	11Cl-PF3OUdS	763051–92-9
Hexafluoropropylene oxide dimer acid	HFPO-DA	13252–13-6
4,8-Dioxa-3*H*-perfluorononanoic acid	DONA	919005–14-4
1*H* , 1*H*, 2*H*, 2*H*-Perfluorohexane sulfonic acid	4:2 FTS	757124–72-4
1*H* , 1*H*, 2*H*, 2*H*-Perfluorooctane sulfonic acid	6:2 FTS	27619–97-2
1*H* , 1*H*, 2*H*, 2*H*-Perfluorodecane sulfonic acid	8:2 FTS	39108–34-4
1*H* , 1*H*, 2*H*, 2*H*-Perfluorododecane sulfonic acid	10:2 FTS	120226–60-0

The SLV validated 11 matrixes in Table 2 for the 30 required PFAS compounds for selectivity, recovery, precision, linearity, and LOQ.

**Table 2.** AOAC SMPR 2023.003 Target matrix and validated matrix

Matrix category	Product validated in study
Produce	Raw carrots
Coffee	Ready-to-drink brewed coffee
Milk (liquid)	Whole milk (3.7% fat)
Dairy powders and plant-based protein powders	Organic plant-based protein powder
Eggs	Whole eggs
Seafood (crustaceans and mollusks)	Frozen shrimp
Fish meat and meat of terrestrial animals (raw, cooked, processed)	Raw tuna filet
Edible offal of terrestrial animals	Beef kidney
Fish oil	Fish oil capsules
Foods for infants and young children (baby food)	Strained pumpkin and sweet potato
Pet food and animal feed	Bagged dry dog food

The method partitions PFAS analytes into acetonitrile and purifies the extract using solid-phase extraction (SPE). Depending on the matrix, buffered QuEChERS reagent is added. Samples containing <80% water generally require addition of water before extraction with acetonitrile (MeCN). Sodium chloride (NaCl) with citrate buffering salt was added to make the analytes more soluble in acetonitrile, and magnesium sulfate (MgSO_4_) was added to improve separation of the MeCN from the rest of the sample. A portion of the MeCN supernatant (upper layer) was removed for cleanup and solvent exchange by column SPE.

Fish oil and edible offal did not require addition of QuEChERS reagents to separate enough supernatant for the SPE cleanup, nor did they require sample concentration. Milk tends to precipitate at the low pH of the citrate buffer and was extracted using an unbuffered QuEChERS reagent. Because of the high lipid content, milk was cleaned up using dispersive SPE (dSPE).

Refer to Figure 1 for the outline of sample preparation protocol in the SLV. After cleanup, the sample was concentrated, if necessary, and analyzed by liquid chromatography/tandem mass spectrometry (LC-MS/MS).

**Figure 1.**
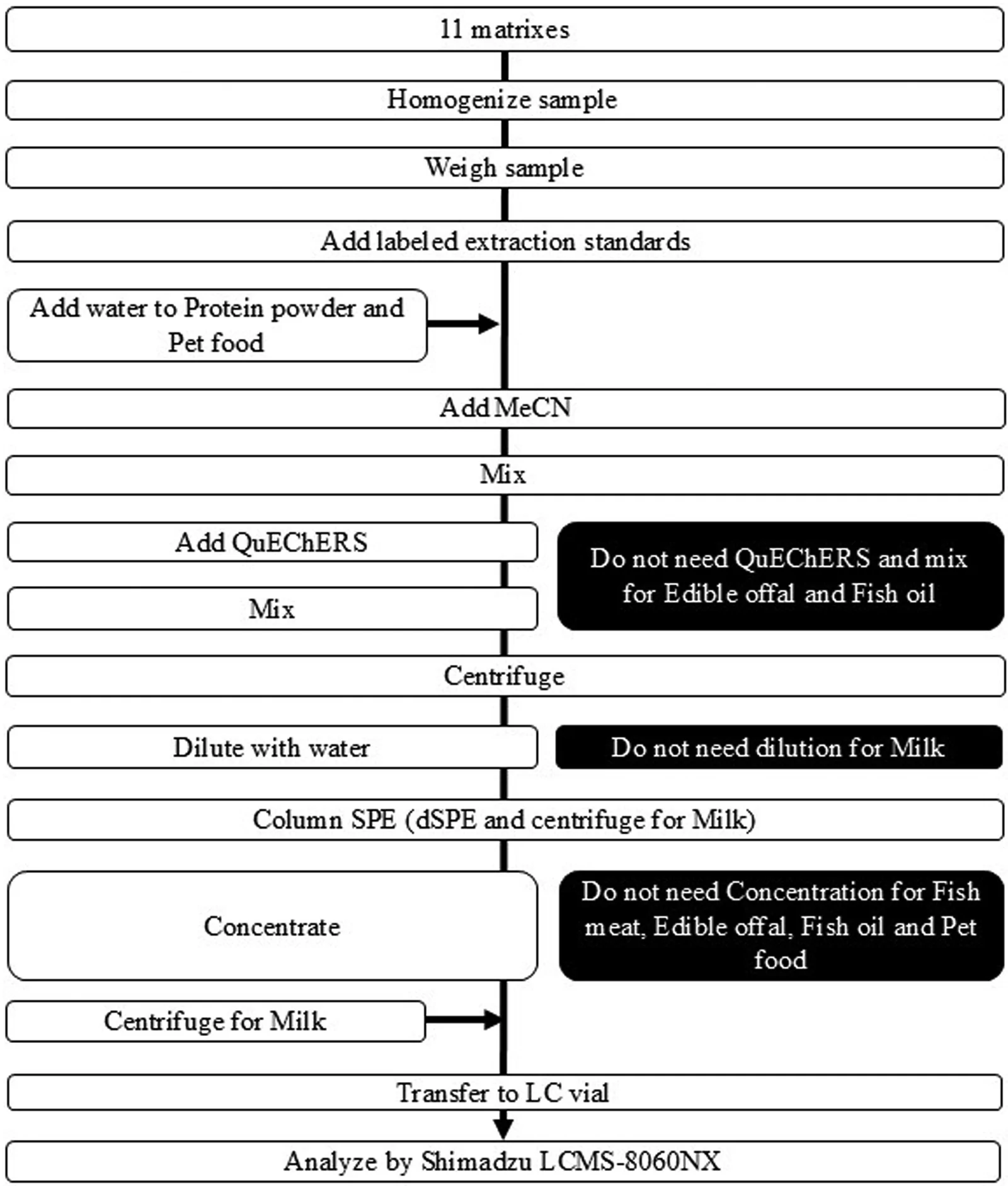
Outline of sample preparation protocol used in the SLV.

Due to potential matrix effects resulting from the LC-MS/MS ionization technique (12–13), matrix-matched calibration standards were prepared and carried out throughout the entire process. Detected analytes were confirmed by retention time matching and ion ratios. Analytes with only one reliable transition were confirmed using high-resolution mass spectrometry (HRMS). Results in μg/kg were calculated directly from the calibration curve using LabSolutions or LabSolutions Insight software. Differences in sample mass were compensated either in the software or by correcting the results manually. The concentration of each of 30 PFAS was determined using the response ratio of the PFAS quantitation transition to that of the relevant labeled isotopic dilution standard. If the target analyte had no corresponding isotope, recovery correction was made using another isotope of similar retention time or recovery. Each sample batch included method blanks, laboratory quality control (QC) samples, and spikes with spike duplicates. QC samples met the criteria provided in the quality control section of the method.

Validation was carried out by spiking each matrix in triplicate at a minimum of three concentrations from or below the estimated LOQ to 20–100 times the LOQ depending on the matrix. Two operators were involved in the validation, with one analyst and instrument in Kanagawa, Japan validating milk, fish meat, baby food, and pet food, and the other analyst in Maryland, USA performing the validation of all other matrixes. To avoid variability between instruments, extensive optimization work was done, resulting in both instruments using the same operating conditions. This ensures the ability to transfer the method between instruments of the same model. Optimization included adjusting for maximum sensitivity of PFOS, PFOA, PFNA, and PFHxS and adjusting chromatography for minimal noise, baseline rise, and to achieve sufficient separation between cholic acids (TDCA, TCDCA, and TUDCA) and PFOS. Non-spiked samples were analyzed to assess PFAS concentration relative to the estimated LOQ. The final LOQ was determined by selecting the lowest concentration of all spike levels that met all SMPR requirements including retention time within 1% of standards, ion ratio ±30%, signal-to-noise ratio ≥3 on the qualifier ion or ≥10 on target analytes without a reliable MS/MS qualifier ion, precision, and recovery. Of all 30 analytes and all 11 matrixes, only two analytes in one matrix (protein powder) resulted in a LOQ high enough that we did not have at least three spike concentrations. All analytes met the LOQ, qualitative, and precision and recovery requirements of the SMPR. Finally, analytes that have no reliable qualifier ion (PFBA, PFPeA, and PFOSA) were confirmed by HRMS using the same LC operating conditions as the determinative method.

## Experimental

### Apparatus

- *Liquid chromatograph/tandem mass spectrometer (LC-MS/MS).*—Nexera liquid chromatograph (LC) system (Shimadzu, Kyoto, Japan) or equivalent LC system, coupled to a LCMS-8060NX triple quadrupole mass spectrometer (MS/MS) (Shimadzu), or equivalent, instrument capable of heated electrospray ionization (ESI) in the negative mode with LabSolutions Software and an autosampler. Separate all analytes using a Shimpack Scepter C18-120, 3 μm, 2.1 × 100 mm analytical column and a Shimpack Scepter C18-120, 3 μm, 2.1 × 50 mm delay column. The C_18_ delay column removes system PFAS contamination, and a bypass valve before the mass spectrometer avoids the introduction of early and late eluting nonanalyte components into the detector. Set up the mass spectrometer with the instrument settings shown in Table 3 and use the specific analyte MRM transitions, CE and quantitation labeled isotope listed in Table 4. These conditions ensure that peak signal-to-noise ratio ≥3 is achieved for the PFAS qualifier ions. PFBA, PFPeA, and PFOSA do not have a reliable qualifier ion of sufficient sensitivity and must meet a signal-to-noise ratio ≥10 on the quantitation ion.
- *Liquid chromatograph/high-resolution mass spectrometer (LC-HRMS).*—Nexera liquid chromatograph (LC) system (Shimadzu), or equivalent, coupled to a LCMS-9030 quadrupole time-of-flight mass spectrometer (QTOF) (Shimadzu), or equivalent. The liquid chromatography separation was performed using the same conditions as described for the LC-MS/MS. The QTOF was operated in ESI negative mode, full scan (150–800 *m/z*), and an event time of 0.6 s. Source conditions duplicated the LC-MS/MS conditions shown in Table 3 except for the desolvation line temperature raised to 250°C, Interface temperature raised to 300°C, and an interface voltage of -3kV.
- *Chromatography data system software.—*Shimadzu LabSolutions with LabSolutions Insight, or equivalent.

**Table 3.** Triple-quadrupole mass spectrometer instrument settings^a^

Instrument parameter	Value
Probe position	+ 2 mm
Nebulizer gas	2 L/min
Heating gas	10 L/min
Drying gas	10 L/min
Desolvation line temperature	200°C
Interface temperature	250°C
Heating block temperature	400°C
Interface voltage	–2 kV
CID gas	270 kPa

a Settings are suitable for the Shimadzu LCMS-8060NX triple-quadrupole mass spectrometer. Settings on alternative instruments may differ.

**Table 4.** MRM transitions, CE and isotopes

Analyte	Quantitation ion			Qualifier ion			Quantitation ion CE	Qualifier ion CE	Quantitation labeled isotope
PFBA	212.9	>	168.8				10		^13^C_4_-PFBA
PFPeA	262.9	>	218.8				8		^13^C_5_-PFPeA
PFHxA	313	>	268.8	313	>	118.9	9	22	^13^C_5_-PFHxA
PFHpA	362.9	>	318.9	362.9	>	168.8	9	16	^13^C_4_-PFHpA
PFOA	412.9	>	368.9	412.9	>	168.8	11	17	^13^C_8_-PFOA
PFNA	462.9	>	418.5	462.9	>	218.6	10	16	^13^C_9_-PFNA
PFDA	512.9	>	468.95	512.9	>	268.55	10	18	^13^C_6_-PFDA
PFUnA	562.9	>	518.95	562.9	>	269	13	17	^13^C_7_-PFUnA
PFDoA	612.9	>	268.6	612.9	>	168.6	19	26	^13^C_7_-PFUnA
PFTrDA	663	>	618.6	663	>	168.5	13	25	^13^C_7_-PFUnA
PFTeDA	713	>	669	713	>	168.5	12	28	^13^C_7_-PFUnA
PFBS	298.8	>	79.8	298.8	>	98.9	33	26	^13^C_3_-PFBS
PFPeS	348.8	>	79.8	348.8	>	98.9	39	30	^13^C_4_-PFHpA
PFHxS	398.8	>	79.8	398.8	>	98.9	44	33	^13^C_3_-PFHxS
PFHpS	448.8	>	79.8	448.8	>	99	49	39	^13^C_6_-PFDA
PFOS	498.8	>	79.8	498.8	>	98.9	54	39	^13^C_8_-PFOS
PFNS	548.9	>	79.8	548.8	>	99	54	43	^13^C_7_-PFUnA
PFDS	598.8	>	99	598.8	>	79.8	45	55	^13^C_7_-PFUnA
PFUnDS	649	>	99	649	>	80	52	55	^13^C_8_-PFOS
PFDoS	699	>	80	699	>	99	54	48	^13^C_2_-PFDoA
PFTrDS	749	>	80	749	>	279.6	54	53	^13^C_7_-PFUnA
PFOSA	498	>	78	498	>	477.95	31	25	^13^C_8_-PFOSA
9Cl-PF3ONS	530.8	>	350.8	532.9	>	352.95	25	25	^13^C_8_-PFOS
11Cl-PF3OUdS	631	>	451	632.9	>	452.95	27	28	^13^C_7_-PFUnA
HFPO-DA	284.8	>	168.8	284.8	>	118.9	7	27	^13^C_3_-HFPO-DA
DONA	376.9	>	250.8	376.9	>	84.9	12	29	^13^C_4_-PFHpA
4:2 FTS	326.9	>	80.9	326.9	>	306.8	31	19	^13^C_2_-4:2 FTS
6:2 FTS	426.9	>	80.8	426.9	>	406.9	36	22	^13^C_2_-6:2FTS
8:2 FTS	526.9	>	506.45	526.9	>	80.55	26	46	^13^C_3_-PFHxS
10:2 FTS	626.9	>	606.7	626.9	>	80.9	27	45	^13^C_7_-PFUnA
**Isotopically labeled internal standards**									
^13^C_4_-PFBA-ES	217	>	172				10		
^13^C_5_-PFPeA-ES	268	>	222.9				8		
^13^C_5_-PFHxA-ES	318	>	272.9				9		
^13^C_4_-PFHpA-ES	367	>	321.9				9		
^13^C_8_-PFOA-ES	421	>	375.9				9		
^13^C_9_-PFNA-ES	472	>	426.9				10		
^13^C_6_-PFDA-ES	519	>	473.8				11		
^13^C_7_-PFUnA_ES	570	>	524.8				11		
^13^C_2_-PFDoA_ES	615	>	569.8				11		
^13^C_2_-PFTeDA_ES^a^	715	>	669.7				13		
^13^C_3_-PFBS-ES	302	>	79.8				32		
^13^C_3_-PFHxS-ES	402	>	79.8				42		
13C_8_-PFOS_ES	507	>	79.8				55		
^13^C_8_-PFOSA_ES	506	>	77.8				33		
^13^C_3_-HFPO-DA-ES	287	>	168.8				7		
^13^C_2_-4:2 FTS-ES	329	>	80.9				26		
^13^C_2_-6:2 FTS-ES	429	>	80.9				35		
^13^C_2_-8:2 FTS-ES^a^	529	>	79.8				44		

a Not used in SLV, but optional.

### Reagents and Materials

All chemicals and solvents were analytical grade unless otherwise specified.

**(a)** *Acetonitrile–MeCN.—*LCMS Grade (Honeywell Cat. No. LC015-4), or equivalent.
**(b)** *Ammonium acetate–NH_4_OAc.—*Powder form (Sigma-Aldrich Cat. No. 73594-25G-F), or equivalent.
**(c)** *Ammonium hydroxide –*NH_4_OH.—(Supelco Cat. No. AX1303-6), or equivalent.
**(d)** *Conditioning reagent 1.—*NH_4_OH—MeOH (1:100 v/v).
**(e)** *Conditioning reagent 2.—*Formic acid–water (1:1000 v/v).
**(f)** *Elution solvent.—*NH_4_OH–MeOH–water (1:90:10 v/v/v).
**(g)** *Formic acid*.—LCMS grade (Fisher Scientific Cat. No. A117-50), or equivalent.
**(h)** *Methanol*.—LCMS grade (Honeywell Cat. No. LC365-4), or equivalent.
**(i)** *Mobile Phase A*.—Add 950 mL water to a glass media bottle. Add 50 mL MeCN and 0.154 g of ammonium acetate. Mix thoroughly.
**(j)** *QuEChERS reagents*.—Q-sep^®^ (Restek Cat. No. 25848), or equally effective.
**(k)** *QuEChERS reagents (unbuffered).—*Supel™Que (Millipore-Sigma Cat. No. 55295-U), or equally effective.
**(l)** *Rinse solution*.—Formic acid–MeOH–water (1:400:600 v/v/v).
**(m)** *SPE (cartridge)*.—SPE Evolute PFAS (Biotage Cat. No. 614-0015-CP), or equally effective.
**(n)** *SPE (dispersive)*.—SupelQue PSA/ENVI-Carb™ Tube (Millipore Sigma Cat. No. 55479-U), or equally effective.
**(o)** *Water.—*LCMS grade (Honeywell Cat. No. LC365-4), or equivalent.
**(p)** *Balance.—*With a repeatability of 0.01 g and a 3200 g capacity (Shimadzu TX 3202), or equivalent.
**(q)** *Centrifuge(s).—*Capable of holding the 50 mL centrifuge tubes or bottles used for extraction and 10–15 mL graduated centrifuge tubes or 2 mL mini-tubes (KOKUSAN Cat. No. H-60R and TOMY Cat. No. MDX-310), or equivalent.
**(r)** *Centrifuge tubes.—*Polypropylene (PP) 50 mL (VWR Cat. No. 89039-656), or equivalent.
**(s)** *Centrifuge tubes.—*Polypropylene (PP) 15 mL graduated (VWR Cat. No. 89039-664), or equivalent.
**(t)** *Reservoir.—*Disposable 25 mL reservoir for 6 mL SPE Cartridges (Supelco Cat. No. 54258-U), or equivalent.
**(u)** *Freezer.—*Capable of continuous operation <–20°C.
**(v)** *Food chopper and/or blender.—*S-blade cutter (AiSTI SCIENCE FST-4000), or equivalent.
**(w)** *Grinder.—*Cryogenic Grinder Knife Mill (Retsch Cat. No. 20.254.0002), or equivalent.
**(x)** *LC vials.—*1.5 mL clear glass vials with cap and septa (Shimadzu Cat. No. 220-97331-30), or equivalent.
**(y)** *Manifold.—*Manifold for SPE with disposable liner (Supelco Cat. No. 57265), or equivalent.
**(z)** *Metal or PP spatula/spoon and funnel.—*For transferring sample into centrifuge tubes.
**(aa)** *Mini-centrifuge tubes.—*2 mL (Eppendorf Cat. No. 022363352), or equivalent.
**(bb)** *Pipet tips.—*LTS 5 mL (RAININ Cat. No. 17001133), or equivalent.
**(cc)** *Repeating or volumetric pipets.—*Capable of accurately transferring 0.5–8 mL solvent.
**(dd)** *Shaker.—*Mechanical shaker (AS ONE Cat. No. AS-1N), or equivalent.
**(ee)** *Solvent dispenser and 1–4 L solvent bottle.—*For transferring 10 mL MeCN per 10 g sample in PP centrifuge tubes or bottles.
**(ff)** *Solvent evaporator (optional).—*(MICROVAP Organomation Cat. No. 11824 or Biotage TurboVap LV), or equivalent.
**(gg)** *Tube.—*1.5 mL for milk (Eppendorf Cat. No. 0030 120.086), or equivalent.
**(hh)** *Vortex mixer.—*Vortex Genie 2 (Scientific Industries Cat. No. SI-0236), or equivalent.

### Standard Solutions

Obtain calibration and labeled extraction standard as mixes from a commercial supplier. Refer to the certificate of analysis for concentrations of each individual analyte.

- *Stock native calibration or spiking solutions.—*Native PFAS precision and recovery standard solution 1000 ng/mL (Wellington Laboratories Cat. No. PFAC30PAR), sodium perfluoro-1-undecanesulfonate 50.0 μg/mL (Wellington Laboratories Cat. No. L-PFUdS), sodium perfluoro-1-tridecanesulfonate 50.0 μg/mL (Wellington Laboratories Cat. No. L-PFTrS), sodium perfluoro-1-dodecanesulfonate 50.0 μg/mL (Wellington Laboratories Cat. No. L-PFDoS), sodium 1H, 1H, 2H, 2H-perfluorododecanesulfonate (10:2) 50.0 μg/mL (Wellington Laboratories Cat. No. 10:2 FTS).
- *4-Mix intermediate standard 1000 ng/mL.—*Dilute ∼100 μL each of the L-PFUdS 50.0 μg/mL, L-PFTrS 50.0 μg/mL, L-PFDoS 50 μg/mL, and the 10:2 FTS 50 μg/mL to 1 mL with MeOH in a PP tube. These concentrations list the salt concentrations. Use the concentration of the analyte provided on the Certificate of Analysis (COA) to calculate the exact volume to add in preparing the standards. Store at –20°C for no more than 6 months. Bring to room temperature before use.
- *Intermediate 30 compound calibration standard 500 ng/mL.—*Combine 500 μL of the native PFAS precision and recovery standard solution 1000 ng/mL and 500 μL of the 4-mix intermediate standard 1000 ng/mL Intermediate solutions in a PP tube. Prepare additional solutions as needed. Store at –20°C for no more than 6 months. Bring to room temperature before use.
- *Intermediate 30 compound calibration standard 100 ng/mL.—C*ombine 100 μL of the native PFAS precision and recovery standard solution 1000 ng/mL and 100 μL of the 4-Mix Intermediate standard 1000 ng/mL Intermediate solutions in a PP tube and add 800 μL MeOH. Prepare additional solutions as needed. Store at –20°C for no more than 6 months. Bring to room temperature before use.
- *Labeled extraction standard.—*Mass-labeled PFAS extraction standards solution 250–5000 ng/mL (Wellington Laboratories Cat. No. MPFAC-HIF-ES). Follow the manufacturer’s instructions for storage.
- *Diluted labeled extraction standard 50–1000 ng/mL.—*Combine 60 μL of the labeled extraction standard and 240 μL MeOH in a PP tube. Prepare additional solutions as needed. Store at –20°C for no more than 6 months. Bring to room temperature before use.

### Sample Preparation

Care must be taken during sampling, subsampling, transferring to other containers, and blending or grinding to minimize contamination and to obtain homogenous samples. A common approach for preparation of food is grinding it with dry ice or liquid nitrogen. This cryogenic grinding makes samples brittle, enabling them to fracture easily, and helps to liberate analyte confined within cell walls. The samples were then stored frozen and thawed to room temperature before test portions were weighed and extracted. Blending is effective in processing large samples relatively quickly. Blenders may be used when only minimal particle size reduction is necessary, or when hand shaking does not adequately mix samples from various portions. For homogenous samples that do not need compositing and with no need for particle size reduction, manual or mechanical shaking is usually sufficient.

- *Produce, eggs, seafood, fish meat, edible offal, and pet food.—*Reduce large portions of samples into fragments by cutting or using an appropriate chopper or blender until the sample gives a consistent texture. Freeze the sample at –20°C and grind with dry ice or liquid nitrogen. Transfer the sample to a suitable container and store at –20°C. Ensure that dry ice has sublimated before weighing test portions.
- *Coffee, milk, protein powders, and fish oil.—*Mix well by shaking the container by hand, and aliquot directly from the container.
- *Baby food.—*Homogenize in a blender, or prepare like produce, depending on the matrix.

### Extraction and Cleanup

Depending on the water content of the matrix, a nominal 10 g test portion typically yields 7–9 mL of MeCN supernatant after centrifugation; however, smaller volumes may be encountered. For samples that do not contain appreciable water, water should be added. The samples may be extracted without the addition of QuEChERS reagents if recovery is acceptable. For the SLV, QuEChERS reagents were not used to extract fish oil or edible offal, and water was added to the pet food and protein powder. All matrixes except for milk were purified using column SPE. For purification by column SPE, the method requires a minimum 1 + 4 dilution of the MeCN with water before column SPE cleanup. Milk was cleaned up using dSPE.

The method may be scaled appropriately to any subsample size shown to be adequately representative of the original sample. We used 10 g test portions extracted in 50 mL PP centrifuge tubes for all matrixes except pet food, which was reduced to 2 g because of the matrix.

We verified that the extraction occurred correctly by confirming that four layers were contained in the tube after the addition and shaking with the QuEChERS reagent. The bottom layer contains excess extraction salt, above it a layer of water, then a layer of sample solids, and finally a top layer of supernatant solution. When the QuEChERS reagent was not used, there were only two layers.

### Preparation of the Matrix-Matched Calibration Curve

Matrix-matched isotope dilution calibration curves were prepared for each matrix validated in the SLV, for a total of 11 matrix matched calibration curves. To make final calculations easier, all standards were added to a known PFAS free matrix (defined as less than 30% of LOQ), calculated as μg/kg, and extracted through the entire process. Extracting the standards enabled concentrations to be calculated by the software directly from the calibration curve. At least four 10.0 ± 0.1 g analyte-free portions of a PFAS-free matrix were weighed into PP centrifuge tubes, one for each of a minimum of three calibration levels and at least one calibration blank (0-standard). For pet food, a 2.00 ± 0.01 g portion was used.

Standards were prepared by adding increasing amounts of standard solution and a midpoint concentration of labeled standard solution to each vial and calculating ng/g. Calibration curves were prepared at concentrations ranging from at or just below the LOQ to an upper concentration that encompassed the expected concentrations of PFAS in the samples, without saturating the detector. For produce, coffee, protein powders, eggs, and seafood, 0.001, 0.01, 0.1, 1.0, and 10 ng/g of each native analyte and 0.05–1.0 ng/g labeled analyte were prepared by adding 0.1, 1.0, 10, 100, and 1000 μL of the100 ng/mL intermediate standard and 10 μL of the 50–1000 ng/mL diluted labeled extraction standard to 10 g test portions of PFAS clean matrix contained in a 50 mL tube. For milk and baby food, a 0.005, 0.01, 0.05, 0.1, 0.5, and 1.0 ng/g of each native analyte and 0.05–1.0 ng/g labeled analyte was prepared by adding 0.5, 1.0, 5, 10, 50, and 100 μL of the 100 ng/mL intermediate standard and adding 10 μL of the 50–1000 ng/mL diluted labeled extraction standard to 10 g test portions of PFAS clean matrix contained in a 50 mL tube. For fish meat, a 0.05, 0.1, 0.5, 1.0, 2.5, and 5.0 ng/g of each native analyte and 0.25–5.0 ng/g labeled analyte was prepared by adding 1.0, 2.0, 10, 20, 50, and 100 μL of the 500 ng/mL intermediate standard and adding 10 μL of the 250–5000 ng/mL labeled extraction standard to 10 g test portions of PFAS clean matrix contained in a 50 mL tube. For pet food, a 0.25, 0.5, 2.5, 5.0, 12.5, and 25.0 ng/g of each native analyte and 1.25–25.0 ng/g labeled analyte was prepared by adding 1.0, 2.0, 10, 20, 50, and 100 μL of the 500 ng/mL intermediate standard and adding 10 μL of the 250–5000 ng/mL labeled extraction standard to 2 g test portions of PFAS clean matrix contained in a 50 mL tube. For fish oil and edible offal, a 0.1, 0.5, 1.5, 5.0, and 15 ng/g of each native analyte and 0.25–5.0 ng/g labeled analyte was prepared by adding 2.0, 10, 30, 100, and 300 μL of the 500 ng/mL intermediate standard and adding 10 μL of the 250–5000 ng/mL labeled extraction standard to 10 g test portions of PFAS clean matrix contained in a 50 mL tube. The concentration of each calibration standard level and each labeled analyte in ng/g was entered into the LabSolutions software, enabling concentrations in the samples to be read directly from the curves. Results were converted to μg/kg to be consistent with reporting requirements of SMPR (1 ng/g = 1 μg/kg). *See*  Table 5 for concentrations of calibration standards used in the SLV.

**Table 5.** Calibration standard concentrations used in the SLV (ng/g)

Matrix	Cal 1	Cal 2	Cal 3	Cal 4	Cal 5	Cal 6
Produce	0.001	0.010	0.100	1.00	10.0	
Coffee	0.001	0.010	0.100	1.00	10.0	
Protein powders	0.001	0.010	0.100	1.00	10.0	
Eggs	0.001	0.010	0.100	1.00	10.0	
Seafood	0.001	0.010	0.100	1.00	10.0	
Milk	0.005	0.010	0.050	0.100	0.500	1.00
Baby food	0.005	0.010	0.050	0.100	0.500	1.00
Fish meat	0.05	0.10	0.50	1.00	2.50	5.00
Pet food^a^	0.25	0.50	2.50	5.00	12.5	25.0
Edible offal	0.100	0.500	1.50	5.00	15.0	
Fish oil	0.100	0.500	1.50	5.00	15.0	

a 2 g sample for pet food; all others 10 g.

Standards for each matrix were extracted as described under extraction and cleanup (Extraction Methods 1–4) and analyzed. Weighted isotope corrected linear calibration curves were used for quantitation of samples.

For Extraction Methods 1–4, refer to Table 6 for sample weight, additional water, volume of MeCN, QuEChERS, supernatant volume, dilution water volume, volume to SPE, elution solvent volume, volume to concentrate, and final volume. Test portions were spiked in triplicate at the concentrations shown in Tables S1–S11 for each matrix. An unspiked portion was also analyzed in triplicate.

**Table 6.** Extraction method

	Method 1			Method 2		Method 3	Method 4
Conditions	Produce, coffee, eggs, seafood	Fish meat	Baby food	Protein powders	Pet food	Edible offal, fish oil	Milk
Sample, g	10	10	10	10	2	10	10
Additional water, mL	No	No	No	20	10	No	No
MeCN, mL	10	10	10	10	10	10	10
QuEChERS (Q-Sep)	Yes	Yes	Yes	Yes	Yes	No	Yes (Supel QuE)
Supernatant, mL	6	7.5	8	2	8.5	4	8
Dilution water, mL	24	30	32	24	34	16	Not applicable
Volume to SPE, mL	30	35	35	26	40	15	8 (dSPE)
Elution solvent, mL	5	5	5	5	5	5	Not applicable
Concentration, mL	2 → 0.4	No	5 → 1	2 → 0.4	No	No	6 → 1
Final volume, mL	0.4	5	1	0.4	5	5	1

### Extraction Method 1 (Produce, Coffee, Eggs, Seafood, Fish Meat, and Baby Food)

This extraction, based on the EN 15662 method (14), was used on matrixes that contain >80% water. For dry materials, refer to Method 3.

For each matrix, test portions were weighed as indicated in Table 6, transferred into 50 mL PP centrifuge tubes, and MeCN added. Samples were mixed by hand or vortexed for 1 min, and the contents of one Q-sep QuEChERS reagents were added to each tube. The tubes were shaken vigorously by hand for 1 min with 3–5 tubes at once in each hand, using the elbows and shoulders more so than the wrists, ensuring that the solvent interacted well and that crystalline agglomerates were broken up sufficiently during shaking. Samples were centrifuged for 5 min at 4000 rpm. A volume of supernatant (upper layer) was transferred to a PP tube and diluted 4 times its volume with water. Using the volume indicated in Table 6, the sample was transferred to an SPE column and eluted with 5 mL elution solvent and concentrated to 1 mL. Fish meat did not need concentration to meet the LOQ requirements of the SMPR. Samples were transferred to an LC vial for analysis.

### Extraction Method 2 (Protein Powders and Pet Food)

This extraction was based on the EN 15662 method; however, since the water content is low, water was added before extraction.

For each matrix, test portions were weighed as indicated in Table 6, transferred into 50 mL PP centrifuge tubes, water added, mixed, and then the MeCN added. Samples were extracted as described in Method 1. Pet food did not need concentration.

### Extraction Method 3 (Edible Offal and Fish Oil)

This extraction is based on the EN 15662 method; however, the SLV demonstrated that addition of QuEChERS reagents was not necessary.

For each matrix, test portions were weighed as indicated in Table 6, transferred into 50 mL PP centrifuge tubes, water added, mixed, and then the MeCN added. Samples were extracted as described in Method 1. QuEChERS reagent and sample concentration were not needed.

### Extraction Method 4 (Milk)

This extraction used unbuffered QuEChERS reagents and dSPE containing PSA and ENVI-Carb. During the SLV, this method was shown to be effective for PFAS in milk with up to 3.7% fat.

Test portions were weighed as indicated in Table 6, transferred into 50 mL PP centrifuge tubes, and 150 μL of formic acid and the MeCN was added. Samples were shaken vigorously by hand for 10 s, and then the contents of one unbuffered QuEChERS reagent—Supel Que—was added. Each tube was mixed for 10 s by hand and for 5 min on a mechanical shaker, and then centrifuged for 5 min at 4000 rpm. Supernatant was transferred to a separate 50 mL PP tube, and a Supel Que PSA/ENVI-Carb was added. Samples were shaken by hand for 10 s for 5 min on a mechanical shaker and then centrifuged for 5 min at 4000 rpm. Supernatant was transferred to a 15 mL tube and concentrated. Once the sample was reconstituted, the entire sample was transferred to a 1.5 mL tube, centrifuged at 15000 rpm, and then transferred to an LC vial for analysis.

### SPE Column Cleanup

SPE Vacuum manifold and Evolute SPE cartridges with disposable reservoirs were assembled according to the manufacturer’s instructions. SPE cartridges were conditioned with 5 mL conditioning reagent 1. Cartridges were not allowed to go dry during the conditioning step. Once conditioning reagent 1 reached the surface of the packing, 5 mL of conditioning reagent 2 was added. As soon as conditioning reagent 2 reached the surface of the packing, sample solutions were added. Each tube was rinsed with 5 mL water and then washed with 5 mL of rinse solution. When the rinse solution reached the top of the packing, the stopcock was closed.

A 15 mL tube was placed under the SPE cartridge and 5 mL of Elution Solvent added, and the stopcock was opened to collect the eluate.

### Sample Concentration

For matrixes that needed greater sensitivity, the volume of eluate indicated in Table 6 was transferred to a clean tube and evaporated at 50–60°C to just dry under nitrogen gas, then reconstituted in methanol–water (90:10, v/v), according to Table 6.

### Determination by LC-MS/MS and Confirmation by LC-HRMS

The instrument was equilibrated until pressure was stable and verified leak-free. Before injection of samples, several solvent blanks were injected to ensure there is no contamination. A suitable volume of sample or standards, 5–10 μL depending on the matrix, was injected into the Shimadzu LCMS-8060NX. Ion ratios were set using the midpoint standard with a tolerance of ±30%. Retention times were verified to be within 1% of the average of the standards in the calibration curve. The sample sequences included a null blank (methanol), a 0-standard plus a minimum of three calibration levels, the unspiked sample triplicates, and the spiked sample triplicates. Because PFBA, PFPeA, and PFOSA only have one MS/MS transition (PFOSA has a weak qualifier ion transition that was not reliable in some matrixes), LC-HRMS was used to confirm the presence of these analytes. For the SLV, extracted ion chromatograms were generated for the exact mass of PFBA and PFPeA in an egg matrix. For PFBA (212.9792 *m/z*), PFPeA (262.9760 *m/z*), and PFOSA (498.9534 *m/z*), acceptance limits were set at 5 ppm.

### Calculations

Quantitation was based on analyte peak area and internal standard (IS) area ratios plotted versus analyte concentration. Weighted linear fits were used in the SLV. For calibration matrixes, we used only matrixes with PFAS no greater than 30% of the required LOQ; during the SLV, no target analytes were detected in any of the matrixes we tested above 30% of the LOQ. The LabSolutions software enabled automatic calculation of accuracy of standards (residual) and recovery of spiked samples, after inputting the expected concentrations. The software also accounted for dilutions or mass corrections when sample weights were not all the same. Since calibration standards are extracted the same as samples, the concentration of analyte in unknowns was determined directly from the calibration curve with no additional calculation.

Some of the PFAS calibration stock standards were listed on the Certificate of Analysis (COA) in concentrations as the salt. When preparing calibration standards for PFAS, we converted these compounds to concentration of that PFAS as an anion and adjusted the volume to use when preparing standards using the equation below:


V = V1 × (C1/C2)

where V = volume of stock or intermediate stock standard to use in μL; C_1_ = concentration listed as the salt; C_2_ = concentration of the analyte as the anion.

Example: the L-PFUdS, is listed as 50 μg/mL of salt. From the COA, the anion is 45.5 μg/mL. The amount to add of L-PFUdS in the *4-Mix Intermediate standard 1000 ng/mL* is calculated as follows:


V = 100 μL × (50 μg/mL/45.5 μg/mL) = 110 μL

Concentration (C_cal_) of each analyte or labeled analyte in the calibration standards or spiked test portions was calculated using the equation below:


Analyte in ng = Cs × (V/1000)

Ccal = Analyte in ng/W

where Cs = concentration of the analyte in the stock or intermediate stock standard used in ng/ml; V = volume of stock or intermediate stock standard to use in μL; C_cal_ = concentration of the calibration standard in ng/g; and W = weight of the test portion in grams.

Example: for fish oil and edible offal, to prepare a 0.1 ng/g of each native analyte add 2.0 μL of the intermediate 500 ng/mL standard to 10 g test portions and calculate as follows:


Analyte in ng = 500 ng/ml × (2μL/1000μL/ml) = 1 ng

Ccal = 1ng/10 g = 0.1 ng/g

To prepare a 0.25 ng/g of the labeled analytes, we added 10 μL of the labeled extraction standard 250–5000 ng/mL (assume the concentration of the example labeled analyte is 250 ng/mL) to 10 g test portions and calculated as follows:


Ccal (ng/g) = 250 ng/ml × (10 μL/1000μL/ml) × 1/10 g = 0.25 ng/g

The concentration for each C_cal_ calibration level for all analytes and the spiked concentration for each spike was entered into the LabSolutions software. Because standards and samples were extracted with the same initial weight and final volume, the concentrations in samples are determined directly (in ng/g) from the calibration curve.

Variability in sample weights can be corrected as follows; however, this correction was also available in the software. During the SLV, each test portion was carefully weighed so no correction was needed.


Corrected Sample Concentration (ng/g) = Cc × (W/Ws)

where C_c_ = concentration of the analyte calculated from the calibration curve in ng/g; Ws = actual weight of the test portion in grams; W = weight of test portion used in calculating the concentration of the calibration standards in grams.

Example for a 10.5 g test portion with 10 g used to calculate concentration of standards that detected 1.5 ng/g analyte from the calibration curve:


Corrected Sample Concentration (ng/g) = 1.5 ng/g × 10 g/10.5 g = 1.42 ng/g

Report results as μg/kg (1 ng/g = 1 μg/kg).

Recovery for each of the spiked samples were calculated as follows:


$$\mathrm{Recovery}\;\%\;=\;[(C_{F}-C_{U})/C_{A}]\;\times \;100$$

where C_F_ = concentration of analyte in the spiked test portion; C_U_ = concentration of analyte in the unspiked test portion; C_A_ = concentration of analyte added to the test portion.

Repeatability for each of the three triplicates at each concentration was calculated as follows:

RSD_r_ % = (s_x̄_/x̄) × 100

where S_x̄_ = standard deviation of the mean of the spiked test portions in ng/g; x̄ = mean of the concentrations of the test portions in ng/g.

### Data Analysis and Quality Control

**Figure 2.**
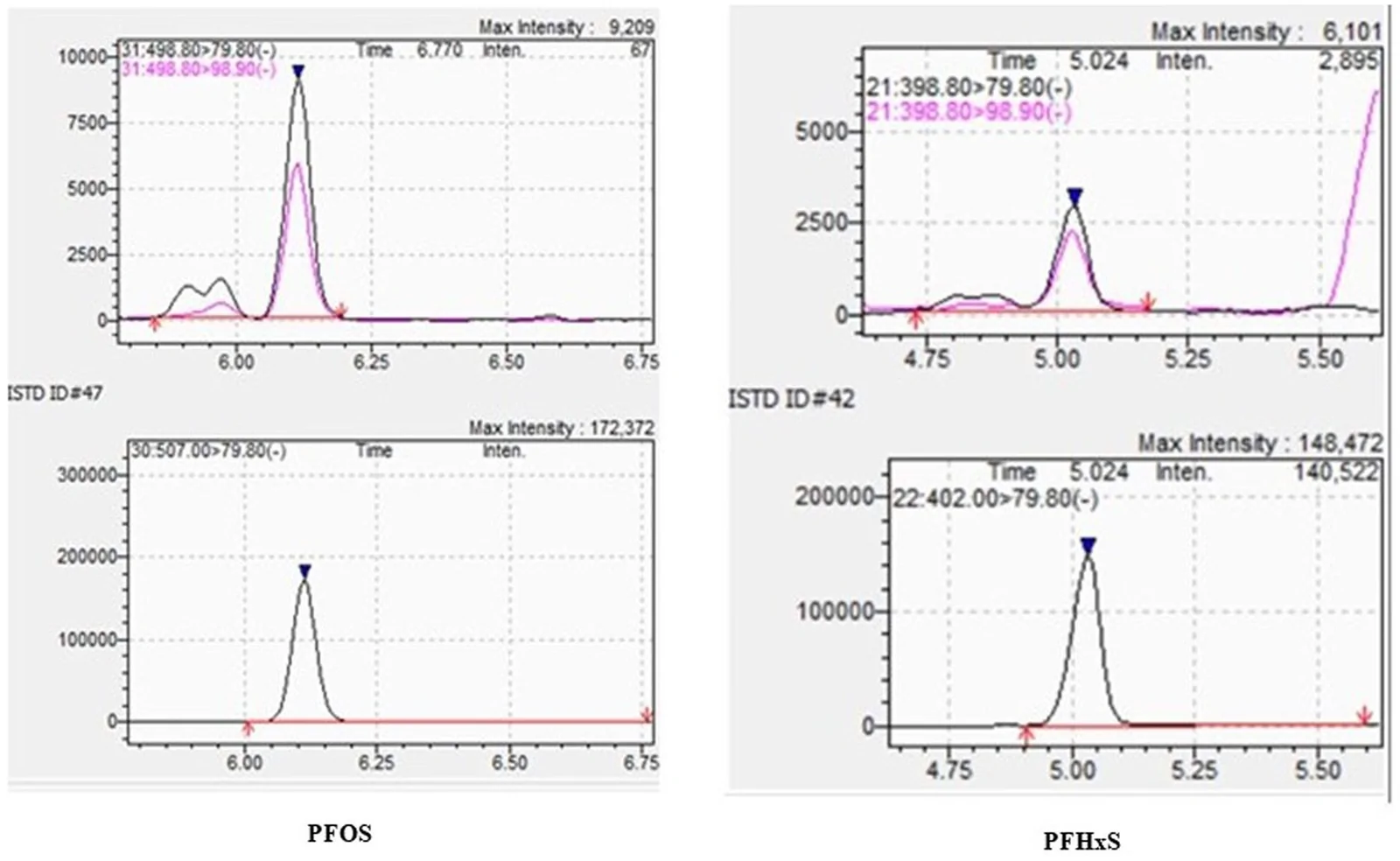
Example integration of PFOS and PFHxS.

The following criteria were used during data collection for verification of the useability of individual results used in the SLV. Per the SMPR, the ion ratio was ±30% or the qualitative identity of all analytes with a confirmation ion except PFOSA. In the matrixes we tested, the ion ratio for PFOSA was inconsistent and did not meet these criteria on all matrixes. For this reason, we concluded the PFOSA ion ratio was unreliable. Retention time of target peaks were within ±1% of the average of the calibration points in the same sequence, unless otherwise noted. Accuracy of the calibration standards and mid-range calibration checks were made by verifying the residuals within ±25% of the expected value using LabSolutions software. We verified that reagent and procedural blanks, including the 0-standard were <30% of the LOQ. PFOS and PFHxS consist of isomeric mixtures. The entire isomeric group was quantified. *See*  Figure 2 for an example integration of PFOS and PFHxS. The SMPR required a check for cholic acid interferences. We obtained sufficient chromatographic separation between the cholic acids (TDCA, TDCDA, and TUDA) and PFOS to ensure no false positives for PFOS if cholic acids were in the samples. Additionally, the ±30% ion ratio for PFOS provides another check for cholic acid interference. We did not detect any interference from cholic acid in the matrixes tested. According to the SMPR, spiked sample recovery must be 80–120% for PFOS, PFOA, PFHxS, and PFNA in eggs, seafood (crustaceans and mollusks), fish meat and meat of terrestrial animals, and edible offal of terrestrial animals with the RSD less than or equal to 20%. Spiked sample recovery must be 65–135% for PFOS, PFOA, PFHxS, and PFNA and all other analytes in all matrixes with the RSD less than or equal 25%. Unless otherwise noted, all results used in the SLV met these criteria.

## Results and Discussion

### Method Development

The PFAS in food method was validated to demonstrate conformity to SMPR for the determination of PFAS in the 11 matrix categories and products shown in Table 2. The validation included optimization of the liquid chromatography (LC) conditions, optimization of mass spectrometry conditions, calibration, interferences, extraction protocols, and matrix studies for recovery and precision. Studies on produce, coffee, dairy powders, eggs, seafood, edible offal, and fish oil at Shimadzu Scientific Instruments Inc. in Columbia, MD, USA, and studies for milk, fish meat, baby food, and pet food were performed at Shimadzu Corp. in Kawasaki, Kanagawa, Japan. Standardizing instrument and chromatography conditions before conducting matrix studies enabled easy transfer of the instrument method between the different locations, instruments, and operators.

### Mass Spectrometer Operating Conditions Study

Mass spectrometer source and ionization conditions were optimized by introducing the mixed PFAS standard into the Shimadzu LCMS-8060NX detector. In these experiments, collision induced dissociation (CID) gas pressure, desolvation line (DL) temperature, drying gas flow rate, heating block temperature, heating gas flow rate, interface temperature, interface voltage, and nebulizer gas flow rate were all tested to maximize the response of the various PFAS, focusing particularly on the EU-regulated PFAS, which are PFOS, PFOA, PFNA, and PFHxS. These experiments were conducted at both locations, in Columbia, MD, USA and in Kawasaki, Kanagawa, Japan. Next, at the Columbia, MD location, we tested two separate settings of heating gas flow rate against the response of PFOS, PFOA, PFNA, and PFHxS. From these experiments, the settings estimated to achieve maximum sensitivity for most of the analytes were chosen. These settings are shown in Table 3.

Multiple reaction monitoring (MRM) and collision energies (CEs) were optimized for each target and labeled analyte transition including secondary, or qualifier, ions if available. Using flow injection (FIA) analysis, standards were introduced into the Shimadzu LCMS-8060NX, and MRM transitions and CEs were evaluated using the Shimadzu LabSolutions software MRM optimization tool. Each MRM transition and CE is entered into a database file that can be readily accessed for inclusion of compounds into a method. For this study, the transitions of the compounds were already available in the database. The MRM transitions and CEs used for this study are shown in Table 4.

### Liquid Chromatography Operating Conditions Study

For our initial chromatographic separation experiments, we tested a Shim-pack Velox SP-C18 (100 mm × 2.1 mm × 1.8 μm) analytical column with a Shim-pack Scepter C18-120 (50 mm × 2.1 mm × 1.9 μm) delay column. This column enabled an approximate 10 min run; however, it did not provide baseline resolution between the branched and linear PFOS isomers. Next, we evaluated a Shim-pack Scepter C18-120 (100 mm × 2.1 mm × 1.9 μm) with a Shim-pack Scepter C18-120 (100 mm × 2.1 mm × 1.9 μm) delay column and a Shim-pack Scepter C18-120 (100 mm × 2.1 mm × 1.9 μm) with a Shim-pack Scepter C18-120 (50 mm × 2.1 mm × 1.9 μm) delay column. Each of these combinations exceeded the upper pressure limits of the columns. Finally, we tested a Shim-pack Scepter C18-120 (100 mm × 2.1 mm × 3 μm) as the analytical column with a Scepter C18-120 (100 mm × 2.1 mm × 3 μm) delay column and a Scepter C18-120 (100 mm × 2.1 mm × 3 μm) analytical column with a Scepter C18-120 (50 mm × 2.1 mm × 3 μm) delay column. We found each of these two latter combinations to be acceptable; however, we used the 50 mm delay column for the SLV because of its lower back pressure. *See*  Figure 2 for an example of a PFOS and a PFHxS peak showing baseline resolution between the linear and branched isomers using this column combination. Other chromatographic conditions including flow rate, gradient, and injection volume were evaluated. The final conditions chosen are shown in Table 7. These conditions maximize separation and sensitivity while minimizing noise and systemic contamination.

**Table 7.** Liquid chromatography instrument setting^a^

Instrument parameter	Value
Mobile Phase A	2 mM ammonium acetate in 5/95 = MeCN–water
Mobile Phase B	MeCN
Analytical column	Shimpack Scepter C18–120, 3 µm, 2.1 × 100 mm
Delay column	Shimpack Scepter C18–120, 3 µm, 2.1 × 50 mm
Oven temperature	40°C

Mobile phase gradient			
		Mobile phase composition	
Time, min	Flow rate, mL/min	A%	B%
0	0.3	80	20
10	0.3	0	100
10.01–12	0.6	0	100
12.01–15	0.3	80	20

a Settings are suitable for the Shimadzu Nexera Liquid Chromatograph. Settings on other instruments may vary.

### Selectivity Study

**Figure 3.**
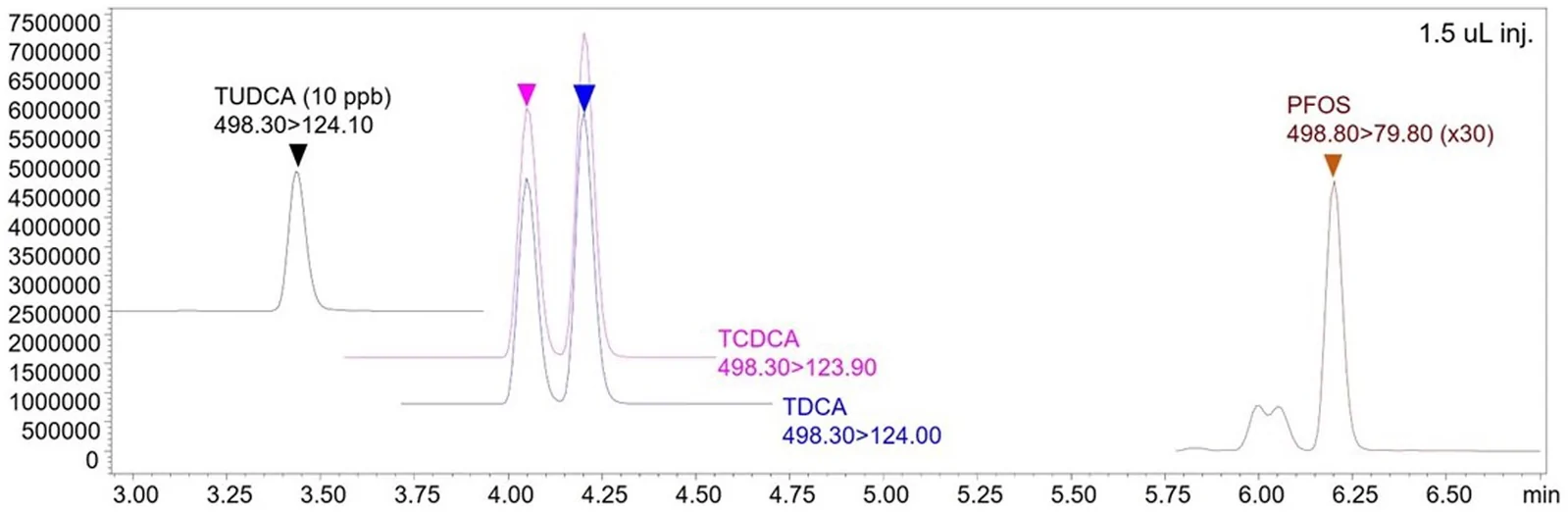
Chromatogram shows nearly 2 min separation of cholic acids and PFOS.

The high selectivity of tandem mass spectrometry in MRM mode minimizes specific co-elution interferences; however, cholic acids taurodeoxycholic acid (TDCA), taurochenodeoxycholic acid (TCDCA), and tauroursodeoxycholic acid (TUDCA) have one similar transition with PFOS *m/z* 499→80. To determine PFOS in extracts that contain, or may contain, cholic acid, baseline separation must be achieved, or the method extraction must include a separation step to remove cholic acid interference. The method may be set up to include monitoring of the secondary *m/z* 499→124 cholic acid transition or use high-resolution mass spectrometry; however, we obtained a near 2 min separation between the cholic acids and PFOS, adequate to ensure no overlap, as demonstrated in Figure 3. Absence of interference for PFOS by cholic acids was confirmed using the ion ratio.

### Extraction Optimization

Generally, the procedure is based on the QuEChERS protocol (15). Samples were spiked in triplicate at three or more concentrations, a suitable portion of labeled extraction standard added, and extracted. Validation of the Qsep reagent on carrots was performed by Shimadzu Corp. earlier (16).

**Figure 4.**
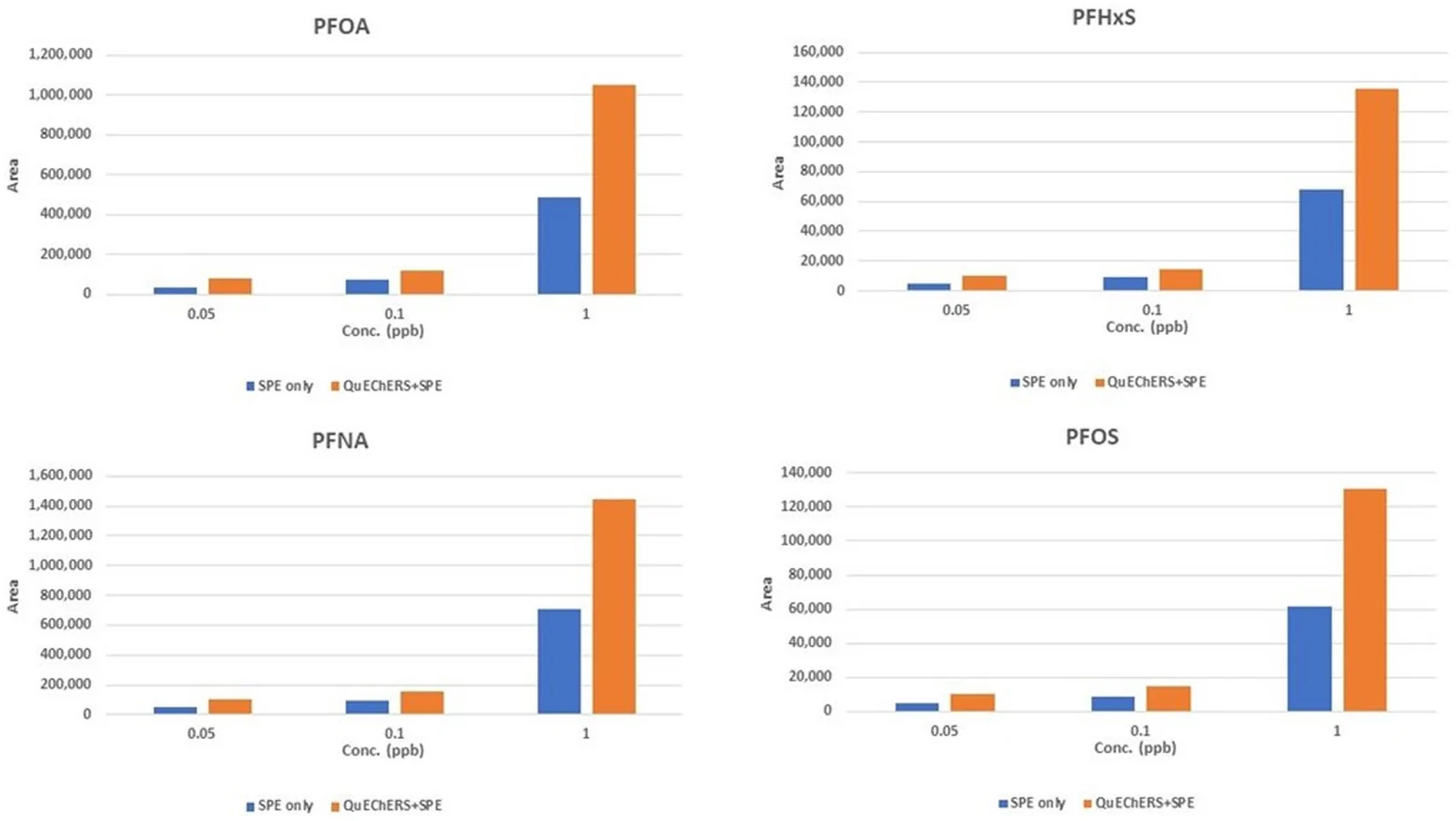
Response of PFOS, PFOA, PFNA, and PFHxS with and without QuEChERS in carrot matrix.

We evaluated analyte response with and without the addition of the QuEChERS reagents. Here we found better or equal response without the QuEChERS reagents in edible offal and fish oil. These data were used in the SLV. For produce, coffee, protein powders, eggs, seafood, fish meat, baby food, and pet food, the response of PFOS, PFOA, PFNA, and PFHxS was low (*see*  Figure 4) without addition of QuEChERS reagents. For these matrixes, the QuEChERS reagents were used in the SLV. For protein powders and pet food, water was added before extraction. All matrixes, except milk, were cleaned up using column SPE. Because of the high fat content in milk, and a tendency for precipitation of proteins, milk was extracted using an unbuffered QuEChERS reagent and cleaned up using PSA and ENVI-Carb dSPE.

### Column SPE Optimization

Two SPE media and three elution schemes each were tested to determine the highest recovery and least interference for the wide range of PFAS required for the method. In these experiments, we spiked a sample of fish meat and tested using the following steps. Load solutions onto an InertSep C18 (500 mg/6 mL) or Evolute Express WAX (150 mg/6 mL). Collect the pass-through elution. Load 5 mL water containing 0.1% formic acid, or 5 mL water, or 5 mL water containing 1% NH_4_OH collecting the eluate. Load 5 mL 10% MeOH containing 0.1% formic acid, or 1% NH_4_OH, or 5 mL water collecting the eluate. Repeat with 10% increases in MeOH until a final concentration of 100% MeOH. Analyze each of the collected eluate solutions with Shimadzu LCMS-8060NX.

**Figure 5.**
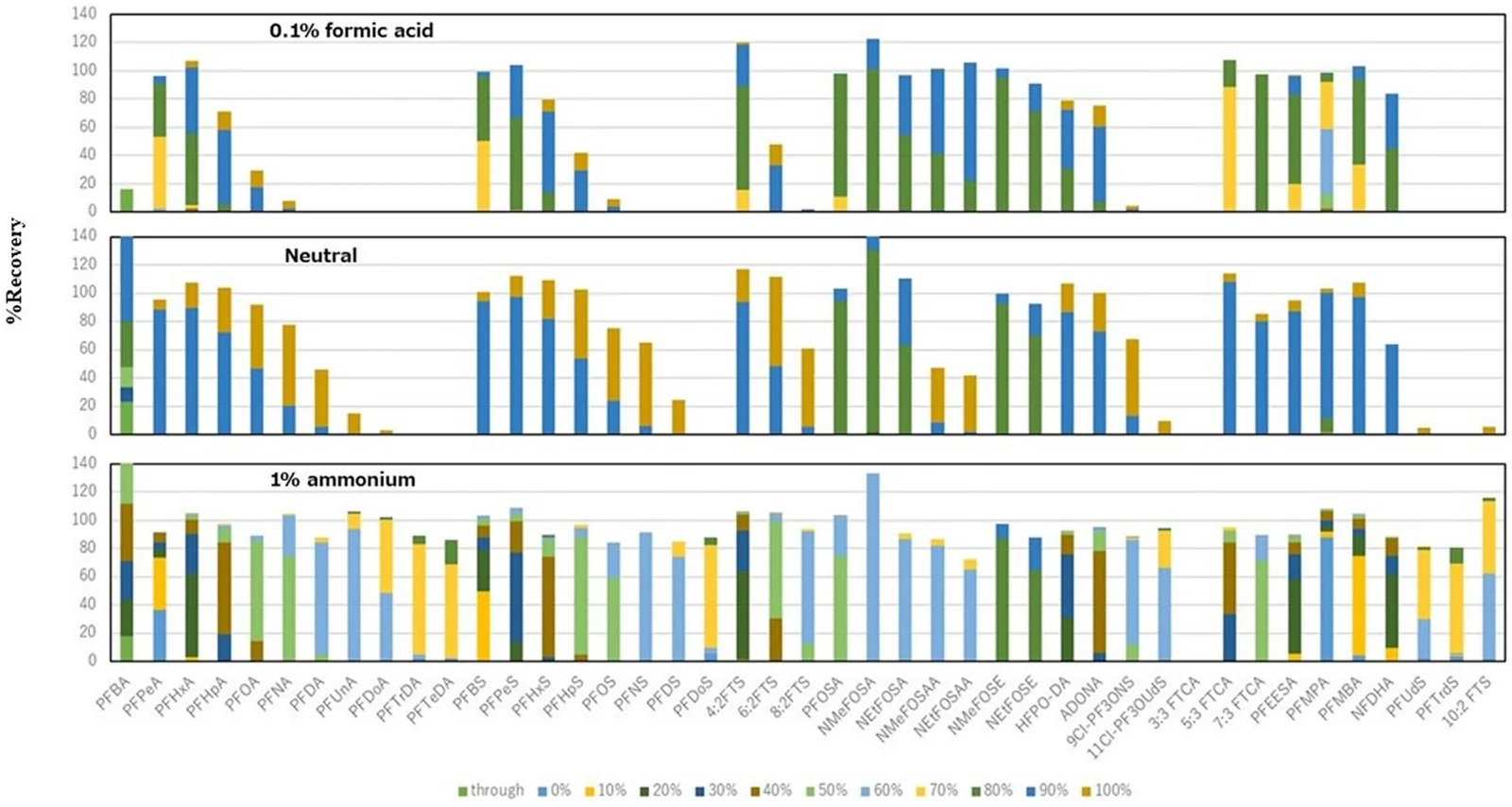
Recovery (%) of target analytes with different elution conditions using InertSep C18 cartridge.

**Figure 6.**
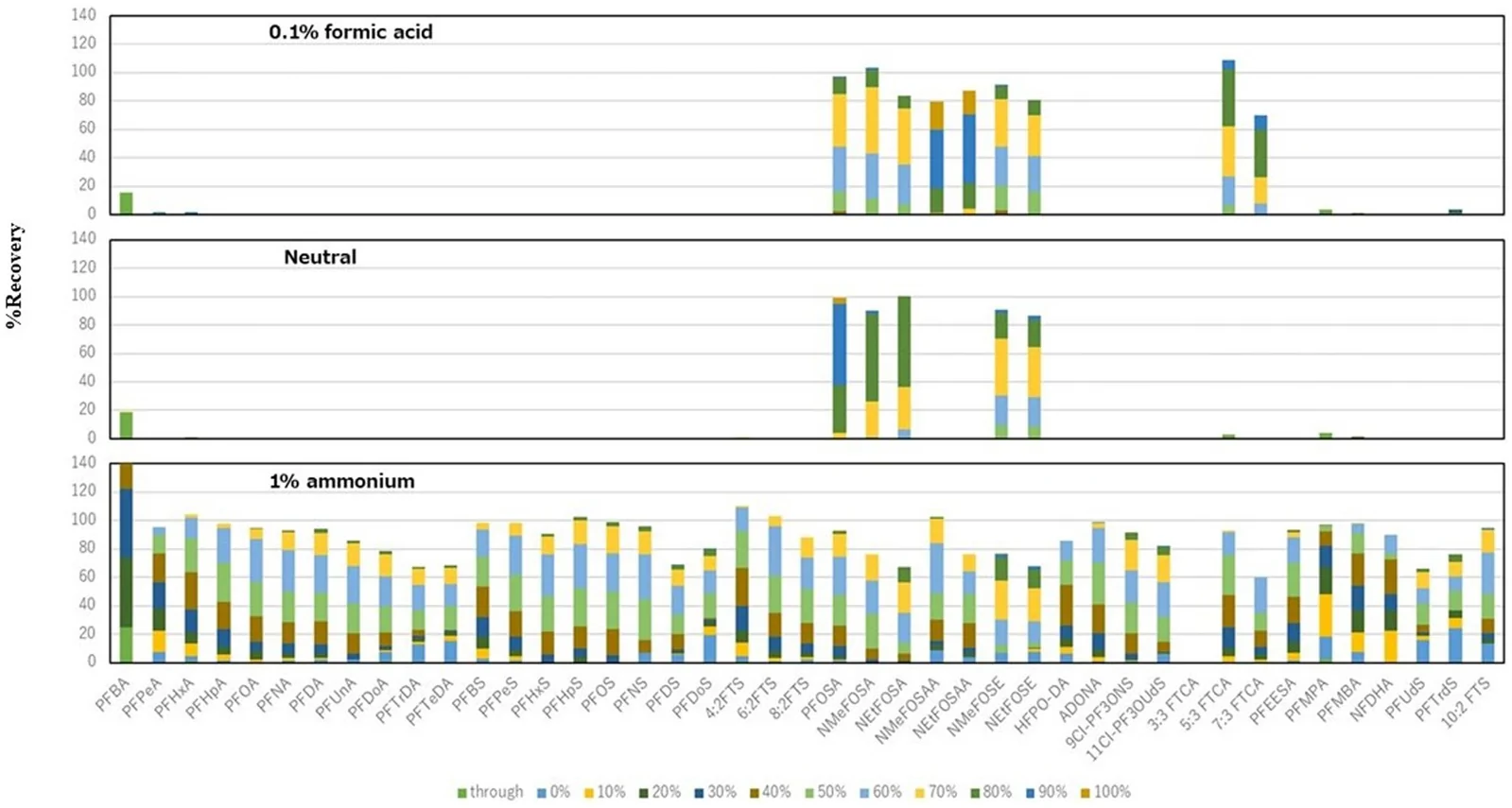
Recovery (%) of target analytes with different elution conditions using Evolute Express WAX cartridge.

We evaluated recovery rates by comparing the peak areas of standard solutions, without isotope correction, of each analyte elution from each column. For both the InertSep C18 (Figure 5) and Evolute Express WAX (Figure 6) cartridges, many of the target compounds were not recovered from the acidic and neutral eluates regardless of MeOH concentration, but all were quantitatively recovered (> 70%) in eluates containing ≥80% MeOH. Therefore, an eluate containing 90% MeOH and 1% NH_4_OH was used for the study. Either the InertSep C18 or Evolute Express WAX could be used; however, for the SLV we chose to use the Evolute Express WAX cartridge.

### Selection of Isotopes for Calibration and Quantitation of Results

The labeled isotopes in Table 4 represent the corresponding unlabeled native analytes. For the native analytes that do not have a respective isotope, we used the isotopes in Table 4 because these were shown in the SLV to have similar recovery to the referenced native analyte. A few ^13^C_2_ labeled isotopes that have a matching native were excluded in favor of non-matching labeled isotopes due to variable recovery in the SLV. For example, because the number of heavy carbon labels in ^13^C_2_-PFDoA-ES is 2, there is an overlap of the naturally occurring ^13^C in PFDoA adding to the ^13^C_2_-PFDoA-ES response. Even though the same amount of ^13^C_2_-PFDoA-ES is added to the calibration samples and the sample or standard, the area ratio becomes inaccurate due to the increasing area of ^13^C_2_-PFDoA-ES as the concentration of PFDoA in increases. Therefore, for labeled isotopes with a small number of labels, we used another labeled isotope as the ISTD if necessary. As mentioned previously, some native analytes that do not have a respective isotope so non-matching isotopes were used. This approach of using a non-matching isotope was not accurate in every matrix tested in the SLV. Thus, isotopic dilution matrix matched calibration curves were used.

### Calibration

**Figure 7.**
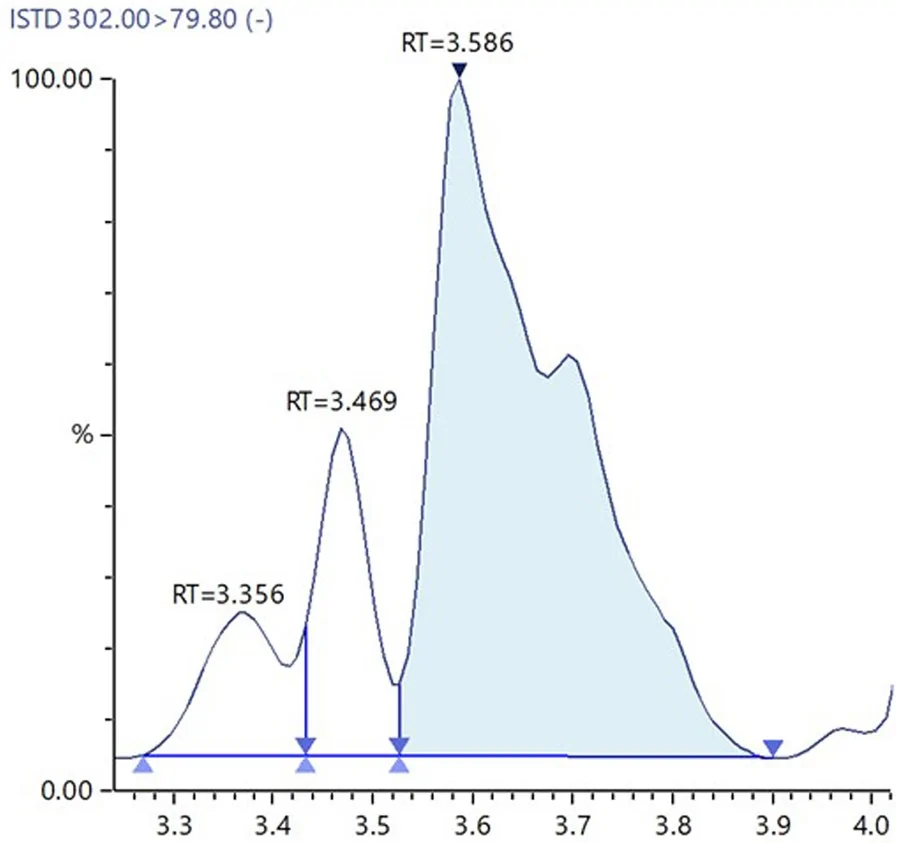
Unknown interference co-eluting with the ^13^C_3_-PFBS isotope in seafood matrix.

A linear model, not forced through zero, isotopic dilution matrix-matched calibration provided the best fit. Matrix-matched calibration standards were processed exactly as samples and ranged from below the SMPR required LOQ to 10–25 μg/kg depending on the matrix. Residuals of each point in the curve were back-calculated and verified to meet ±25% of the expected value. Exact labeled analogs were used as isotope dilution standards, except in a few cases (for example, ^13^C_2_-PFTeDA). In these cases, another isotope was chosen and standardized for all matrixes. The analytes tested and the associated calibration isotopes are shown in Table 4. During the SLV, the seafood matrix (shrimp) contained an unknown interference coeluting with the ^13^C_3_-PFBS isotope (Figure 7), and ^13^C_5_-PFHxA was used. Additionally, ^13^C_2_-PFDOA was used in place of ^13^C_8_-PFOSA for the coffee matrix due to low and variable recovery of the ^13^C_8_-PFOSA standard.

### Matrix Blanks

None of the matrixes evaluated in the SLV contained target analytes >30% of the experimentally determined LOQ.

### Ion Ratios

Reference ion ratios were calculated by dividing the qualifier ion area by the quantitation ion area and multiplication by 100 to obtain a percentage. We set the ion ratio for each analyte using the midpoint calibration standard. Then, we applied a ± 30% limit and compared the actual ratio of the samples with the calculated limit. Only data with passing ion ratios were included in the SLV.

### Calculation of Signal-to-Noise Ratio

The Shimadzu LabSolutions software enabled several methods for the determination of the signal-to-noise ratio. For the SLV, we used a root-mean-square (RMS) approach that auto-selects baseline segments for estimation of noise, and noise was calculated as the standard deviation of the baseline over the transition window. The peak height was then divided by the calculated noise.

### Determination of the LOQ

**Figure 8.**
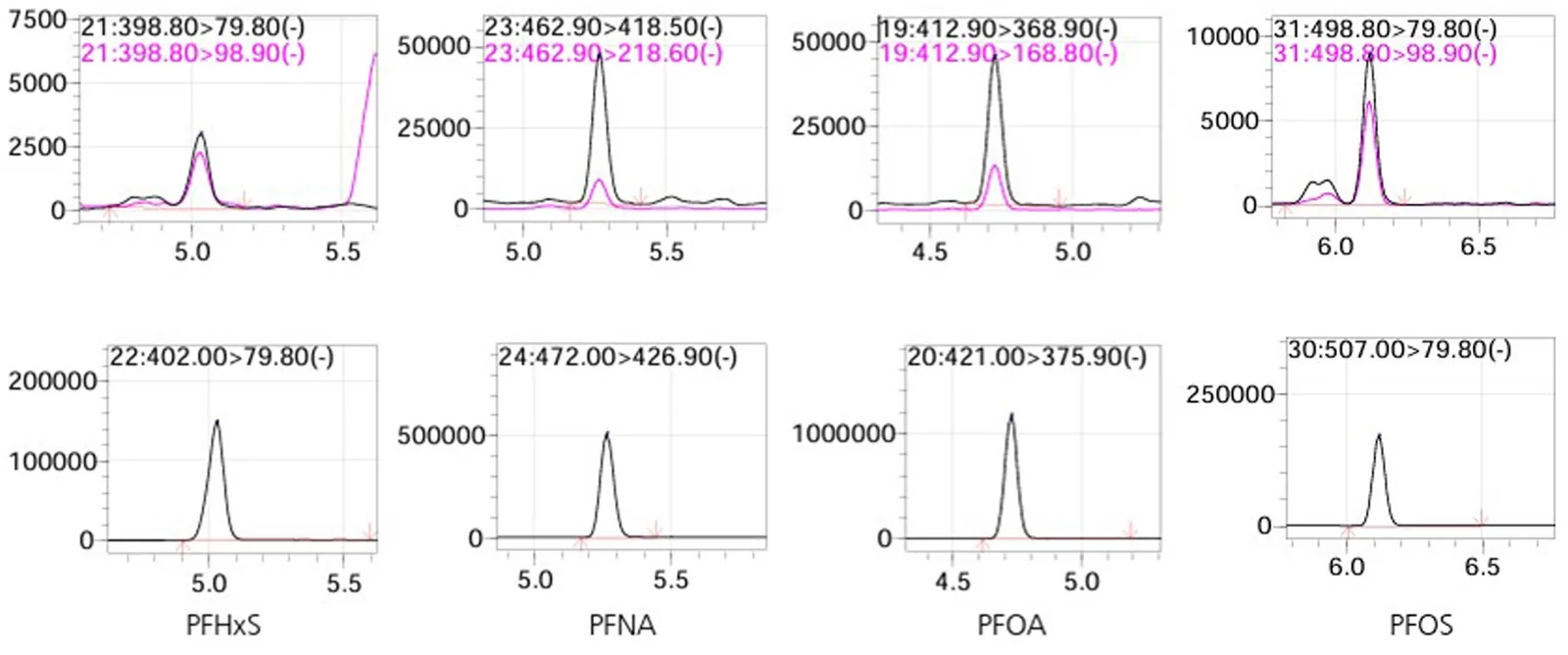
Examples of the lowest 0.0055 µg/kg matrix spike in a carrot matrix for PFHxS, PFNA, PFOA, and PFOS and their corresponding IS.

The LOQ were determined by spiking all matrixes at concentrations at, or below, the required LOQs listed in the SMPR (Table 8). The spiked samples were analyzed in triplicate, and the mean and repeatability standard deviation were calculated. Then, the standard deviation was divided by the mean and multiplied by 100% to calculate the repeatability percent relative standard deviation (RSD). The LOQs (Table 9) for all matrixes and analytes were determined using an Excel worksheet that compared each of the requirements of the SMPR including blank, ion ratio, retention time, recovery, repeatability, and signal-to-noise ratio >3 for the qualifier ion. Retention times for all analytes in all matrixes were matched with the average of the calibration points in the same sequence with a tolerance of 1%, except PFHxA in eggs and PFBA in milk. However, the retention time of these analytes matched their internal standard (*see* S13). The LOQ of PFBA, PFPeA, and PFOSA which did not have reliable qualifier ions was set at the minimum concentration meeting the requirements of the SMPR with a signal-to-noise ratio >10. Figure 8 shows examples of the 0.0055 μg/kg matrix spike in a carrot matrix for PFHxS, PFNA, PFOA, and PFOS and their corresponding IS.

**Table 8.** Required limits of quantification (LOQ)

	LOQ, µg/kg						
Matrix category	PFOS	PFOA	PFNA		PFHxS	PFBA and PFPeA	Other PFAS
Eggs	≤0.3	≤0.3	≤0.3	≤0.3		≤3	≤3
Seafood (crustaceans and mollusks)	≤0.3	≤0.3	≤0.3	≤0.3		≤3	≤3
Fish meat and meat of terrestrial animals	≤0.1	≤0.1	≤0.1	≤0.1		≤1	≤1
Edible offal of terrestrial animals	≤0.4	≤0.4	≤0.4	≤0.4		≤4	≤4
Produce	≤0.01	≤0.01	≤0.01	≤0.01		≤1	≤0.1
Coffee	≤0.3	≤0.3	≤0.3	≤0.3		≤3	≤3
Milk (liquid)	≤0.01	≤0.01	≤0.01	≤0.01		≤1	≤0.1
Dairy powders and plant-based protein powders	≤0.08	≤0.08	≤0.08	≤0.08		≤1	≤0.8
Fish oil	≤0.5	≤0.5	≤0.5	≤0.5		≤5	≤5
Food for infants and young children (baby food)	≤0.01	≤0.01	≤0.01	≤0.01		≤1	≤0.1
Feed	≤0.5	≤0.5	≤0.5	≤0.5		≤5	≤5

**Table 9.** LOQ in µg/kg for each PFAS and all matrixes

Analyte	Produce	Coffee	Milk	Protein powders	Eggs	Seafood	Fish meat	Edible offal	Fish oil	Baby food	Pet food
PFBA	0.055	0.055	0.01	0.055	0.055	0.055	0.1	0.2	0.25	0.1	0.5
PFPeA	0.055	0.0055	0.01	0.055	0.055	0.0055	0.1	0.2	0.25	0.1	0.5
PFHxA	0.0055	0.055	0.01	0.055	0.055	0.0055	0.1	0.2	0.25	0.1	0.5
PFHpA	0.0055	0.0055	0.01	0.055	0.0055	0.055	0.1	0.2	0.25	0.01	0.5
PFOA	0.0055	0.0055	0.01	0.055	0.0055	0.0055	0.1	0.2	0.25	0.01	0.5
PFNA	0.0055	0.0055	0.01	0.0055	0.0055	0.0055	0.1	0.2	0.25	0.01	0.5
PFDA	0.0055	0.055	0.01	0.0055	0.0055	0.0055	0.1	0.2	0.25	0.01	0.5
PFUnA	0.055	0.0055	0.01	0.0055	0.0055	0.0055	0.1	0.2	0.25	0.01	0.5
PFDoA	0.0055	0.055	0.01	0.055	0.0055	0.055	0.1	0.2	0.25	0.01	0.5
PFTrDA	0.0055	0.055	0.01	0.055	0.0055	0.055	0.1	0.2	0.25	0.1	0.5
PFTeDA	0.0055	0.055	0.01	0.055	0.055	0.055	0.1	0.2	0.25	0.1	0.5
PFBS	0.055	0.0055	0.01	0.055	0.0055	0.055	0.1	0.2	0.25	0.01	0.5
PFPeS	0.0055	0.0055	0.01	0.055	0.0055	0.055	0.1	0.2	0.25	0.01	0.5
PFHxS	0.0055	0.0055	0.01	0.0055	0.0055	0.0055	0.1	0.2	0.25	0.01	0.5
PFHpS	0.0055	0.055	0.01	0.0055	0.0055	0.0055	0.1	0.2	0.25	0.01	0.5
PFOS	0.0055	0.055	0.01	0.055	0.055	0.055	0.1	0.2	0.25	0.01	0.5
PFNS	0.0055	0.055	0.01	0.055	0.0055	0.0055	0.1	0.2	0.25	0.01	0.5
PFDS	0.0055	0.055	0.01	0.055	0.0055	0.0055	0.1	0.2	0.25	0.01	0.5
PFUnDS	0.0055	0.055	0.01	0.055	0.055	0.0055	0.1	0.2	0.25	0.1	0.5
PFDoS	0.0055	0.055	0.01	0.055	0.055	0.0055	0.1	0.2	0.25	0.01	0.5
PFTrDS	0.055	0.55	0.01	0.55	0.055	0.055	0.1	0.2	0.25	0.1	0.5
PFOSA	0.0055	0.55	0.01	0.55	0.55	0.055	0.1	0.2	0.25	0.1	0.5
9Cl-PF3ONS	0.0055	0.0055	0.01	0.0055	0.0055	0.0055	0.1	0.2	0.25	0.01	0.5
11Cl-PF3OUdS	0.0055	0.055	0.01	0.0055	0.0055	0.0055	0.1	0.2	0.25	0.01	0.5
HFPO-DA	0.0055	0.055	0.01	0.55	0.0055	0.055	0.1	0.2	0.25	0.01	0.5
DONA	0.0055	0.0055	0.01	0.0055	0.0055	0.0055	0.1	0.2	0.25	0.01	0.5
4:2 FTS	0.055	0.055	0.01	0.055	0.55	0.055	0.1	0.2	0.25	0.01	0.5
6:2 FTS	0.055	0.0055	0.01	0.055	0.0055	0.55	0.1	0.2	0.25	0.01	0.5
8:2 FTS	0.0055	0.55	0.01	0.0055	0.0055	0.0055	0.1	0.2	0.25	0.01	0.5
10:2 FTS	0.0055	0.055	0.01	0.055	0.0055	0.0055	0.1	0.2	0.5	0.01	0.5

### Confirmation of Detections for Analytes With No Reliable Qualifier Ions

**Figure 9.**
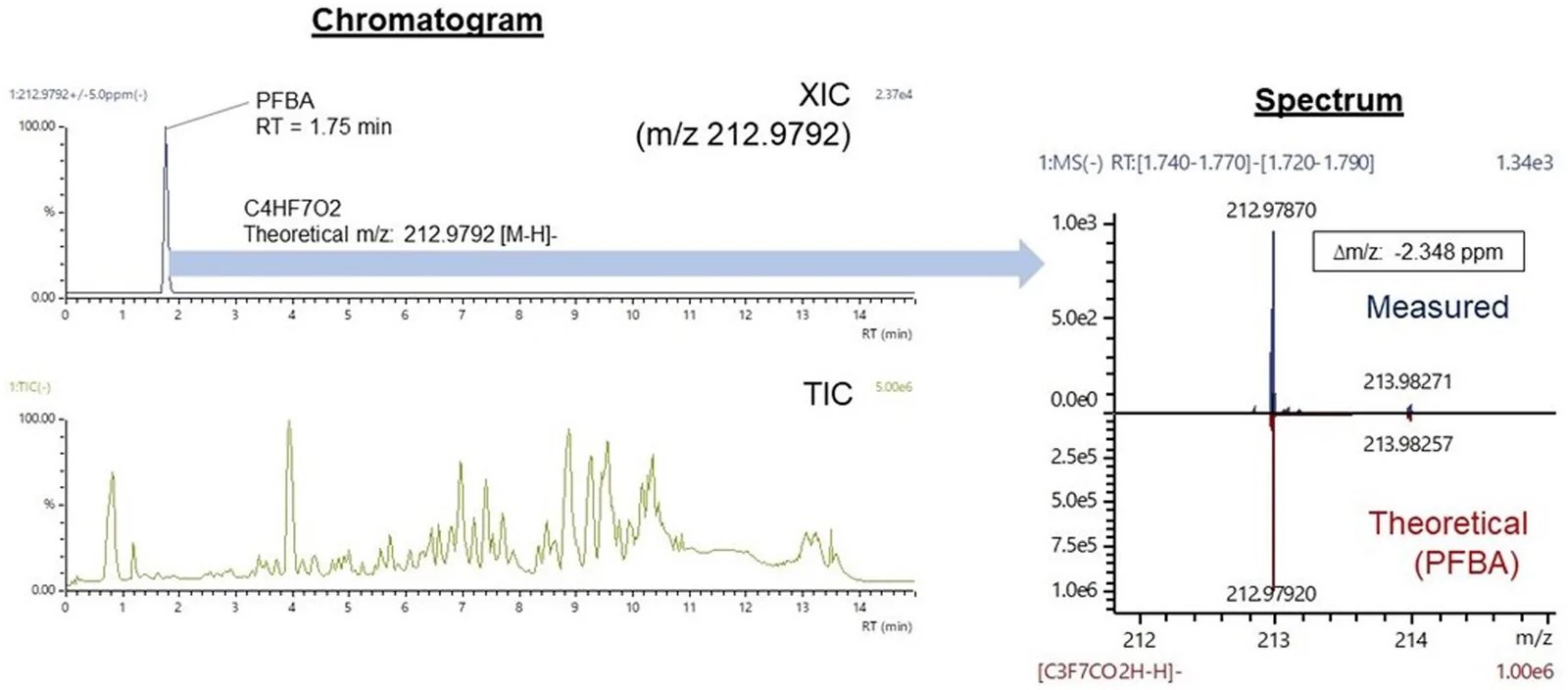
HRMS confirmation of PFBA in egg matrix.

**Figure 10.**
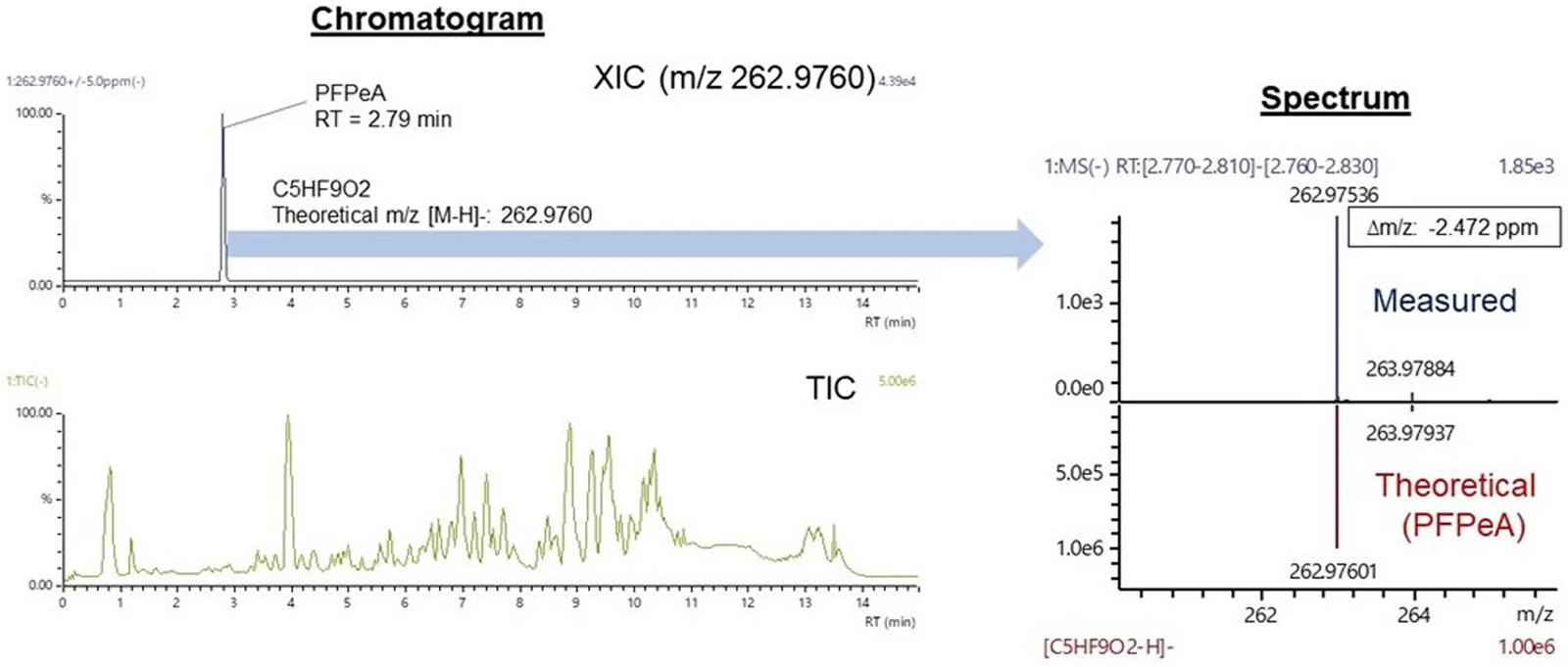
HRMS confirmation of PFPeA in egg matrix.

The SMPR requires that if a second qualifier ion is not available or dependable, to confirm the presence of analytes which are detected above the regulatory limit, or above the LOQ if there is no regulatory limit. To demonstrate the confirmation of analytes with no reliable qualifier ions, we used the Shimadzu LCMS-9030 to analyze spiked extracts of the egg matrix for PFBA (Figure 9) and PFPeA (Figure 10). We recommend similar confirmation if PFOSA is detected.

### Recovery and Repeatability

Blank matrixes and at least three different concentrations ranging from below the SMPR required LOQ to approximately 100 times the estimated LOQ were analyzed in triplicate. Table 10 is a summary of recovery and repeatability of all concentrations for PFOS, PFOA, PFNA, and PFHxS in each matrix. Recovery and repeatability for each analyte at each concentration and each matrix tested are given in Tables S1–11. All analytes in all matrixes except HFPO-DA and PFTrDS in protein powders met the recovery and repeatability requirements of SMPR. Protein powder was spiked at four different concentrations for all analytes. However, for HFPO-DA and PFTrDS the two lower concentrations, both spiked below the required LOQ, exceeded the recovery criteria. Therefore, for HFPO-DA and PFTrDS in protein powders, only two spiked levels were reported.

**Table 10.** Summary of recovery and repeatability of all concentrations for PFOS, PFOA, PFNA, and PFHxS in each matrix

Matrix	Analyte	Average Recovery, %	Highest RSD, %	Lowest RSD, %	Average RSD, %
Produce	PFOS	96	3.77	0.15	1.96
	PFOA	104	3.30	0.42	1.38
	PFNA	102	2.54	0.64	1.30
	PFHxS	94	11.6	1.09	5.48
Coffee	PFOS	91	15.8	1.26	6.80
	PFOA	98	2.62	0.58	1.55
	PFNA	94	4.97	3.24	3.99
	PFHxS	101	12.4	1.16	4.26
Milk	PFOS	98	6.54	1.37	3.50
	PFOA	100	11.4	1.02	5.10
	PFNA	103	0.93	0.62	0.80
	PFHxS	98	10.8	1.42	5.36
Protein powders	PFOS	103	16.8	2.49	7.83
	PFOA	110	4.40	2.10	3.03
	PFNA	108	10.5	0.61	3.98
	PFHxS	107	6.02	1.10	3.22
Eggs	PFOS	97	5.28	0.31	2.46
	PFOA	101	6.19	1.10	2.94
	PFNA	105	7.91	0.54	3.03
	PFHxS	102	19.2	1.09	5.70
Seafood	PFOS	98	5.28	0.99	2.72
	PFOA	103	5.89	0.58	2.19
	PFNA	103	6.77	0.52	2.64
	PFHxS	96	17.6	0.23	5.35
Fish meat	PFOS	99	1.85	0.62	1.13
	PFOA	98	1.51	0.84	1.28
	PFNA	100	2.72	1.12	2.03
	PFHxS	99	6.21	2.09	3.84
Edible offal	PFOS	105	6.94	1.80	4.00
	PFOA	111	5.29	2.34	3.46
	PFNA	107	6.30	2.55	3.93
	PFHxS	97	6.29	1.72	4.21
Fish oil	PFOS	95	5.46	2.60	4.23
	PFOA	103	4.88	1.11	2.68
	PFNA	104	3.36	1.27	2.29
	PFHxS	98	4.83	0.51	2.68
Baby food	PFOS	93	6.56	0.06	3.04
	PFOA	100	3.07	0.38	1.58
	PFNA	100	5.13	1.88	2.92
	PFHxS	98	7.45	2.15	3.60
Pet food	PFOS	95	6.84	1.55	3.91
	PFOA	102	2.68	0.42	1.30
	PFNA	101	0.93	0.65	0.77
	PFHxS	99	3.02	2.07	2.56

## Conclusions

The LC-MS/MS method described has been successfully validated and provides rapid, precise, and quantitative analysis of 30 PFAS in produce, coffee, milk, protein powders, eggs, seafood, fish meat, edible offal, fish oil, baby food, and pet food, meeting the LOQ, recovery, and repeatability requirements of AOAC SMPR 2023.003.

## CRediT Author Statement

Toshiya Matsubara (Data curation [Lead], Methodology [Equal], Validation [Equal], Writing—review & editing [Equal]), Yusuke Mizuno (Project administration [Equal], Writing—review & editing [Equal]), Eishi Imoto (Data curation [Equal], Methodology [Equal], Validation [Equal], Writing—review & editing [Equal]), Nozomi Maeshima (Data curation [Equal], Methodology [Equal], Validation [Equal], Writing—review & editing [Equal]), Manami Kobayashi (Methodology [Equal], Supervision [Equal], Writing—review & editing [Equal]), Okiyuki Kunihiro (Supervision [Equal], Writing—review & editing [Equal]), and William Lipps (Methodology [Lead], Project administration [Lead], Supervision [Lead], Writing—original draft [Lead], Writing—review & editing [Lead])

## Supplemental Information

Supplemental information is available on the *J. AOAC Int.* website.

## Supplementary Material

qsag014_Supplementary_Data
